# Assessing the impact of the COVID-19 infodemic on older people in the region of Crete: Maria Papadakaki

**DOI:** 10.1093/eurpub/ckac131.366

**Published:** 2022-10-25

**Authors:** K Vagionaki, M Papadakaki, E Petelos, C Lionis

**Affiliations:** 1 Department of Social Work, LaHeRS & PHC-HMU, Hellenic Mediterranean University, Heraklion, Greece; 2 Clinic of Social and Family Medicine, University of Crete, Heraklion, Greece

## Abstract

The COVID-19 pandemic has been accompanied by an unprecedented infodemic, a key global public health challenge. Older people are more susceptible to COVID-19 and to misinformation, with WHO indicating the need for research on how people process and manage information in physical and digital environments to better understand how this phenomenon affects individuals. A qualitative study was developed in to explore the infodemic impact on elderly groups (eligible beneficiaries, aged 60-75 y, users of social services in the Heraklion Regional Unit, Crete Greece). Preliminary results indicate low trust levels for information delivered online and through media. Physicians remain the most trusted and preferred source of information, yet participants questioned their expertise level given the various issues emerging during the pandemic. Most of them report poor quality, incomplete information and point out the misleading role of media pluralism. Key topics of concern include the origin of SARS-CoV-2 and the safety and effectiveness of COVID-19 vaccines. The majority strongly believes that coronavirus is of lab origin, as they have read on the Internet. They are also susceptible to fake news and myths surrounding COVID-19 vaccines. Many of them also suffer from “pandemic fatigue”, i.e., information overload, preferring not to further follow COVID-19 updates. Fear about their vulnerability was the major factor mentioned regarding scientific evidence, stating they prefer information to be conveyed clearly and in an understandable manner. Some exchange information with relatives and friends whilst others prefer not to discuss COVID-19, unless they consider the other person knows more than they do. Nevertheless, they all believe they make the best choices on how to protect their health, whilst having mixed feelings about the manner and content of communication received both in physical and digital environments.

**Key messages:**

• COVID-19 care for the elderly should encompass infodemic management elements, incl. information presented using simple terms, from updated and regularly verified sources, ideally in printed form.

• Health and social care professionals’ training should include media discourse, compassionate care, and motivational communication techniques rather than directional speeches of coercion and fear.

